# The International Medical Congress

**Published:** 1867-11

**Authors:** Edmund Andrews

**Affiliations:** Paris


PARIS, Sept. 1, 1867.

TO THE EXAMINER:

The discussion on tuberculosis, to which I referred in my last letter, brought out very contradictory opinions, which are at present irreconcilable.

Mons. VILLEMIN has made elaborate observations and experiments which lead him to believe that tuberculosis is as much a specific disease as syphilis or variola, and that it may be taken by contagion and propagated by inoculation. Mons. LEBERT, on the contrary, thinks that there is nothing specific about tubercle, and that its elements are purely inflammatory products. He has made a series of experiments, by which he believes he has proved that tubercle may be produced by injecting into the blood various products of disease, and even mineral substances, such as mercury and carbon. These particles being carried to the lungs by the pulmonary artery, become lodged in the capillaries mechanically, and acting as points of irritation produce tubercles, inflammatory in their real nature, and having all the microscopic and pathological characters of the tubercles of consumption.

Between these conflicting doctrines, it seems we must rest, and wait for more light. The general drift of sentiment in the Congress was, that there is no peculiar anatomical or histological element in tubercle, whatever may be true of its diathesis, or of its virus, if any exists.

On the second day, among other topics, came up that of menstruation, as affected by climate, race, etc. Some elaborate statistics were produced upon the subject of the average age at which menstruation commences among different people. M. Lagneau gave the following classification of some 15,000 observations:

| Race. | Average age of first menstruation. | | |
|---|---|---|---|
| North Germans, | 16 years, | 9 months, | 16 days. |
| English, | 14 " | 11 " | 2 " |
| French, | 15 " | 1 " | 21 " |
| Southern Asiatics, | 12 " | 11 " | 17 " |

Mr. Robert Cowie, of the Shetland Islands, sent a paper on the "*Prolonged Menstrual Life in the Shetland Islands, and its Relation to Longevity,*" in which he shows that the Shetland women retain that function very late in life, the average time of the turn of life with them being from 50 to 54 years. By comparison with Scotland, he shows that this late retention of the menstrual function is also accompanied with an increased longevity. He asserts that the age of commencing menstruation does not differ from that of other parts of the kingdom.

On the third day, Professor Polli, of Milan, known for his experiments on the antiseptic effects of the sulphites in the inferior animals, read a paper on the use of these articles as medicines in septic diseases, and detailed the Italian experience in their use. Both he and the whole Congress seemed to be in blessed ignorance that this matter had been tested and discussed several years ago in Chicago, and that to Dr. FISHER, Dr. DAVIS, and to the CHICAGO MEDICAL EXAMINER, belonged the credit of publicly establishing the practical value of the remedies. If the European savants would regularly take and read some of the American medical journals, they would often find that ideas which are quite new to them have been discussed for years in America.

On the fourth day, was discussed the very important topic of the accidents which follow surgical operations, such as erysipelas, hospital gangrene, and pyæmia.

It is frightful to contemplate the destruction of life in European hospitals by these diseases, and to perceive that it is kept up by the gross mismanagement of even their best and most eminent men.

The great murderous sin of European hospitals is want of ventilation. They are afraid of pure air, and, consequently, pyæmia is a fearful surgical scourge. Mr. GOSSELIN, at the Hopital de la Pitie, innocently reports that he has an epidemic of erysipelas regularly, almost every winter (at the season when the weather causes the windows to be closed), but seemed to have no suspicion that it might be prevented with ease. He also reports, with the utmost coolness, that of the cases which originate in the hospital every third patient dies. I think this is perfect butchery, and I cannot help comparing it with the better practice in Chicago, where, in Mercy Hospital, deaths from pyæmia and erysipelas have become an almost unknown thing, being completely banished by the simple expedient of opening wide the windows, and keeping every surgical patient, night and day, in a current of pure air. Prof. PAGET, of London, has recently experimented in the same way, though he does not seem to be aware that we had settled the matter in Chicago, and published it years ago. His report confirms my experience. He says that the most efficient preventive of pyæmia, is to place the patient in a constant current of pure air, an announcement which ought to strike like a peal of thunder on the ears of European surgeons. The effect of foul air in

Paris hospitals is such that they do not dare to close wounds by first intention. I have seen them fill the space between the flaps of an amputation with charpie, as they used to do in the dark ages, and the risk of pyæmia is so great as to render many valuable operations wholly unjustifiable.

The surgeons represented in the Congress generally admitted that ventilation favored escape from pyæmia, but the idea rested in their heads in a very sleepy, foggy manner. Not one of them seemed to be aware of the fact that this cause of death can be completely removed by the means of fresh air. The papers which were read showed that the authors were blindly fumbling about in the materia medica, to find some medicament which would enable them to combat pyæmia without pure respiration. Some used a solution of perchloride of iron, applied to the flaps of amputated cases; others applied solutions of carbolic acid; others employed various measures, all very useful and valuable in themselves; but deadly blunders were committed in this respect, *viz.:* they all invited the profession to rely on these secondary measures, instead of *perfecting the ventilation of the hospitals.*

On the fifth day, came up the famous question, "Is it possible to propose to the various governments efficacious measures for restraining the propagation of venereal diseases?"

This question received all sorts of elucidations except clear ones. Those most interested in the discussion, were evidently of the number of those who desired a system of licensed prostitution. Some of the statistics presented were horribly erroneous, as I happened to know from personal investigations, and, on the whole, the discussion of the real question was loose, wandering, and inconclusive.

After the papers were read, verbal discussion upon the subject commenced. The veteran author on syphilis, RICORD, was in the chair. In a few minutes the hot blood of the disputants got the mastery over parliamentary order, the original question was forgotten, and the speakers plunged headlong into a dispute whether syphilitic inoculation was to be commended or not. M. RICORD, though chairman, took an active part in the contest, and pushed his opponents with a relentless vigor that was refreshing to behold, but not very parliamentary for a presiding officer.

M. AUZIAS-TURENNE had advocated "syphilization," as the grand remedy and preventive. M. RICORD rose and said, that at the first publication of the plan of syphilization, he had demanded that the author should test the innocence and excellence of his practice by a trial of it on his own person. He had never yielded to that demand. Now, if M. AUZIAS-TURENNE wished that they should believe in syphilization, let him have the courage to syphilize himself.

*Auzias-Turenne.*—"Syphilization must follow the road of all other scientific questions. It is to be judged by the facts in the annals of science, and not by the measure of my courage or cowardice."

*Ricord.*—"I ask no more than that he should believe his own doctrine. If syphilization is a truth, it follows that soft chancre is to hard chancre what vaccinia is to variola. AUZIAS-TURENNE would then be the JENNER of this kind of pox, and we ought to erect statues to him. Once more, then, I say, if AUZIAS-TURENNE believes in syphilization, let him syphilize himself. Enough of patients have paid their health, and even their lives, for the pretended benefits of syphilization."

*A Member of the Congress.*—[Shouting at the top of his voice.] "I am a physician. I have been syphilized, and I am perfectly healthy."

*Another Member.*—"Why don't AUZIAS-TURENNE do it?"

*The First Member.*—"Because he has not renounced marriage. As for me, I have renounced it. What father or mother would give their daughter to a syphilized man?" [Laughter.]

*Ricord.*—"On the contrary, syphilization being such a safeguard, syphilized men ought to be in great demand for husbands." [Laughter.]

*Auzias-Turenne.*—I have offered, in vain, to make experiments before a commission; they demand that I shall experiment upon myself. I refuse upon my dignity. I have no desire to put myself at the disposition of M. RICORD, to satisfy his curiosity and to serve as the butt of his jokes. I demand that the question be treated scientifically, before a scientific assembly."

Thus the dispute went on, until the hour of adjournment. At a subsequent session, the fight was renewed. Finally, the fact that they had wandered entirely away from the topic of governmental management of syphilis, dawned upon their minds, and the subject was closed by appointing a commission to address the governments on the subject. The following gentlemen were named as the committee: Messieurs HEBRA, Vienna; SCITZ, Munich; CROCQ, Brussels; SECO BALDOR, Madrid; GALLIGO, Florence; PALASCIANO, Naples; OWRE, Christiana; BARBOSA, Lisbon; FRERICHS, Berlin; HUEBBENET, Kiew; FORDYCE BARKER, New York; WILSON JEWELL, Philadelphia; UPHAM, Boston; R. R. MACILVAINE, Cincinnati; HINGSTON, Montreal.

The facts brought out before the Congress on the subject of syphilization, may be briefly summed up as follows:

Prof. BOËCK, of Sweden, treats secondary syphilis by numerous and long-repeated inoculations of the matter of soft chancre. The plan is this: Several inoculations are made at once upon the patient. When the sores are produced, another set is inoculated, and the first ones destroyed by caustic. Thus crop after crop of chancroids is produced, through a period of several months. At length, after an average of about three hundred and forty-five inoculations, a tolerance is produced, and the patient is found incapable of having any more chancroids. He is then pronounced "syphilized" and cured. During this long treatment, the secondary symptoms generally, or at least frequently, disappear.

Against this treatment, it is urged that some patients have been inoculated to complete insusceptibility to soft chancre, and yet have *failed* to be cured of their secondary symptoms; that thoroughly syphilized women have proved that they were not cured, by bearing syphilitic infants. As to the disappearance of secondary eruptions, it is urged that four months is a long time, and that in such a period these eruptions often recede from view, spontaneously.

In a few instances, the most disastrous results, and even loss of life, have followed the treatment. The discussion showed that the opinion of the Congress was decidedly opposed to this practice.

A banquet was held at the Grand Hotel, by such of the members who chose to subscribe for the purpose. About 200 subscribed. The feast opened with great eclat, and good feeling. M. BOUILLAUD was in the chair. Several short, pithy addresses were read, an awkward way of delivery in dinner speeches, but, still, the time went merrily on. In the midst of the festivities, word was brought that the great surgeon VELPEAU had just died. This announcement cast a gloom over the assembly, which silenced its mirth and rendered the remainder of the evening a melancholy occasion.

VELPEAU'S death was rather sudden, as he had been on duty only a few days before, in the hospital. I went the rounds of his wards with him, and saw several of his operations. He was, however, very old, and really superannuated. He held on to his public position too long for his own reputation. His prescriptions and operations, however, were reasonably good, but his wards were the worst ventilated of any that I saw in Paris, and had a strong stench of foul air in every part of them.

At one of the evening sessions of the Congress, M. MILLIOT exhibited, upon a cat and a dog, a new method of physical diagnosis of tumors, etc., in the visceral cavities. His plan is to illuminate very powerfully the interior of the stomach, rectum, bladder, or vagina by electric light, and then to observe the translucency of the abdomen, on the same principle that we test the translucency of the scrotal tumor to diagnose hydrocele. For this purpose, he introduces into the stomach, rectum, etc., small glass tubes, containing an arrangement of platina wires connected with an electric apparatus. In this way, he produces a powerful interior electric light, which renders the abdomen translucent, like a hydrocele. He hopes to use it for the diagnosis of ovarian cysts, but has not yet brought it to any practical usefulness.

At the close of the Congress, the venerable President pronouced a brief farewell address, and declared the sessions finally closed. EDMUND ANDREWS.

